# Factors associated with longitudinal MDS-UPDRS III score trajectories in early-stage Parkinson’s disease

**DOI:** 10.3389/fnins.2026.1759090

**Published:** 2026-02-20

**Authors:** Wen Zhou, Duan Liu, Tian-fang Zeng, Qing-qing Xia

**Affiliations:** West China School of Medicine, Sichuan University Affiliated Chengdu Second People's Hospital, Chengdu Second People’s Hospital, Sichuan University, Chengdu, Sichuan, China

**Keywords:** machine learning, Movement Disorder Society - unified Parkinson’s disease rating scale part III score, Parkinson’s disease, Parkinson’s progression, markers initiative, trajectories analysis

## Abstract

**Background:**

Parkinson’s disease (PD) exhibits significant clinical heterogeneity, particularly in motor symptom progression. This study aims to identify distinct trajectories of motor progression in PD and explore associated predictive factors.

**Methods:**

Data were obtained from the Parkinson’s Progression Markers Initiative (PPMI) database on [2025-3-25]. Motor symptom severity was measured using the MDS-UPDRS III scores. Latent class trajectory analysis was used to identify distinct progression patterns. Multinomial logistic regression and machine learning models were used to evaluate predictors.

**Results:**

Three distinct motor progression trajectories were identified: slow progression (38%), moderate progression (55.9%), and rapid progression (6.1%). Compared to the slow progression group, a higher baseline MDS-UPDRS III score was strongly associated with both moderate (OR = 1.27, 95% CI: 1.23–1.31, *p* < 0.001) and rapid progression (OR = 1.49, 95% CI: 1.43–1.57, *p* < 0.001). Lower serum albumin levels also significantly increased the likelihood of moderate (OR = 0.95, 95% CI: 0.91–0.99, *p* = 0.014) and rapid progression (OR = 0.89, 95% CI: 0.81–0.98, *p* = 0.016). Additionally, higher baseline BMI (per 5 kg/m^2^ increase) was associated with greater odds of moderate (OR = 1.19, 95% CI: 1.01–1.41, *p* = 0.042). Finally, each 1-unit lower mean striatum specific binding ratio (SBR) reduced the odds of moderate progression by 32% compared with the slow-progression group (OR = 0.68, 95% CI: 0.46–0.99, *p* = 0.044). Machine learning analysis confirmed the predictive importance of these factors, with the Random Forest model achieving an AUC of 0.950.

**Conclusion:**

Baseline motor severity, dopaminergic imaging, nutritional status, and body weight are key predictors of motor progression in PD. These findings highlight the potential for early risk stratification and personalized management strategies.

## Introduction

Parkinson’s disease (PD) is a complex, progressive neurodegenerative disorder characterized by a diverse array of motor manifestations, including tremor, bradykinesia, and rigidity, as well as non-motor features such as cognitive impairment, mood disorders, and autonomic dysfunction. The annual incidence of PD ranges from 5 to over 35 per 100,000 individuals, with a notable rise in prevalence from the sixth to the ninth decades of life (Twelves et al., 2003). Notably, PD is distinguished by significant clinical variability, with marked differences in the progression patterns of the disease among patients (Marras and Lang, 2013; Skrahin et al., 2024). This heterogeneity is not only reflected in the clinical symptoms but also in the speed and patterns of disease progression. While some patients experience stable symptoms over extended periods, others may swiftly deteriorate to severe disability.

Emerging evidence suggests that various factors may underpin these distinct prognosis in PD (Moretti et al., 2017; Rajput et al., 2017). However, prior research has predominantly focused on identifying factors associated with the presence, different subtypes and severity of PD symptoms (Elfil et al., 2021; Still et al., 2024; Zhou et al., 2025a, 2025b). A notable gap remains in our understanding of the factors that influence different trajectories of motor symptom progression. This gap is critical because identifying such factors could significantly enhance our ability to predict disease course, tailor therapeutic interventions, and improve patient outcomes.

Given the clinical heterogeneity of PD and the significant impact of motor symptom progression on patient outcomes, this study aims to explore factors associated with different motor symptom progression trajectories in PD. By addressing this gap in the literature, we hope to provide valuable insights for clinical management and contribute to the development of more effective, personalized treatment plans.

## Methods

### Participant selection and study grouping

**Figure 1:**
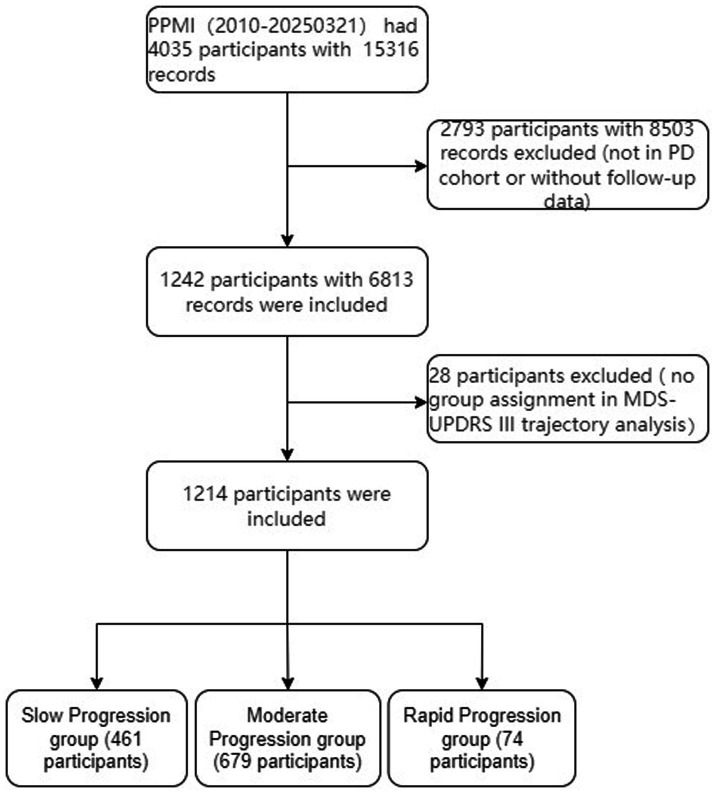
Flowchart of the study cohort.

Data used in the preparation of this article were obtained on [2025-3-25] from the Parkinson’s Progression Markers Initiative (PPMI) database,^1^ RRID: SCR_006431. For up-to-date information on the study, visit http://www.ppmi-info.org. The PPMI cohort is a global observational study that longitudinally assesses participants recruited through neurology clinics associated with academic medical institutions across various countries. For our analysis, we included patients with a diagnosis of PD (Figure 1). The initial diagnosis was conducted by movement disorder specialists and on-site investigators and was subsequently reviewed and confirmed by a central consensus committee to ensure diagnostic accuracy. This study included a long-term follow-up of PD patients, with the longest follow-up period reaching up to 14 years.

PPMI studies were conducted in accordance with relevant regulations and guidelines. Written informed consent was obtained from all participants, and written informed consent was given to the subjects. The PPMI was approved by the local Institutional Review Boards of all participating agencies. All research procedures were conducted in accordance with the Declaration of Helsinki of 1964 and its subsequent amendments.

### Measurements and definitions

To investigate the longitudinal trajectories of motor symptoms in PD patients, we utilized the Movement Disorder Society-Unified Parkinson’s Disease Rating Scale Part III (MDS-UPDRS Part III) scores, which comprehensively assess the severity of motor symptoms. The MDS-UPDRS Part III scores were obtained from the PPMI database, which includes detailed motor assessments conducted at regular intervals. These scores provide a quantitative measure of motor symptom severity, covering aspects such as tremor, rigidity, bradykinesia, and postural instability. The scores were collected during “OFF” medication states to capture the full spectrum of motor symptom variability.

The mean striatum dopamine transporter binding measure (DaTscan striatum) was calculated using DaTscan imaging data, which assesses dopamine transporter density in the striatum. Specifically, the mean striatum measure was derived by averaging the dopamine transporter binding values from the right and left caudate nuclei and putamen, as provided in the PPMI database.

The mild cognitive impairment (MCI) test scores were derived from a composite of cognitive tests administered to assess MCI in PD patients. The indicator for MCI was based on cognitive test scores, where a participant was classified as having MCI if at least two cognitive test scores were more than 1.5 standard deviations (SD) below the standardized mean. The specific cognitive tests included in the assessment were HVLT Total Recall, HVLT Recognition Discrimination, Benton Judgment of Line Orientation, Letter Number Sequencing, Semantic Fluency Test, and Symbol Digit Modalities. Participants were classified as having normal cognition if none of the cognitive test scores exceeded 1.5 SD below the standardized mean, and as having test-based MCI if at least two cognitive test scores exceeded this threshold.

### Statistical methods

The trajectories of MDS-UPDRS III score were analyzed using the latent class trajectory analysis to identify patterns of change over the follow-up period (Nagin et al., 2018). The most appropriate trajectory model was determined through a two-stage model selection process, taking into account factors such as trajectory shape (e.g., linear, quadratic, or cubic) and the number of groups. The number of dimensions and groups were optimized using Bayesian Information Criterion (BIC) values and clinical interpretability. Several models with varying numbers of trajectories (ranging from two to five) were attempted until the best-fitting model was identified. The performance of the candidate models is detailed in Supplementary Table 1. Once the best-fitting model was determined, participants were assigned to MDS-UPDRS Part III score trajectories based on the highest estimated probability of belonging to each group. The discrimination level of the average posterior probability was set at ≥ 0.70. The average posterior probability of participants assigned to each group was sufficiently high (ranging from 0.73 to 0.77), indicating a robust classification. Ultimately, three distinct trajectories were recognized.

The clinical characteristics of participants were summarized, stratified by the three trajectory groups identified based on the MDS-UPDRS III score. To scrutinize the dispersion of continuous variables, we deployed histogram analysis, Q-Q plots, and invoked the Kolmogorov–Smirnov test. Continuous variables adhering to a normal distribution were characterized by their mean ± SD, whereas those exhibiting skewness were depicted by the median (interquartile range, IQR). Categorical variables were elucidated through frequencies and percentages. When comparing continuous variables across the three trajectory clusters, we applied one-way analysis of variance (ANOVA) for normally distributed data and the Kruskal-Wallis H test for data with skewness. For categorical variables, we utilized the chi-square test or Fisher’s exact test, contingent on the data’s suitability, to discern differences among the groups.

To investigate the associations between various factors and the trajectories of MDS-UPDRS III score in PD patients, we conducted a series of statistical analyses after multiply imputing missing covariate values. For each factor of interest, including age, sex, body mass index (BMI), DaTscan striatum, MCI, serum glucose levels, albumin, age at onset of symptoms, and baseline MDS-UPDRS III score, we performed separate multinomial logistic regression analyses (Upton, 1995) to assess its independent association with the MDS-UPDRS III score trajectories. For each candidate predictor we fitted a separate multinomial-logistic regression in which that predictor was the exposure of interest and the remaining eight variables were simultaneously entered as covariates.

To assess the robustness of our findings, we conducted sensitivity analyses using two approaches. First, we excluded all records with missing data for the covariates and re-performed the correlation analysis to verify the stability of the results. Second, to further validate the results over a different time span, we utilized data from the first 10 years of follow-up to re-run the trajectory analysis and correlation analysis.

To investigate the role of various clinical indices in predicting the trajectories of MDS-UPDRS III scores in PD patients, we employed machine learning algorithms (Supplementary Figure 1). Prior to predictive modelling, we consolidated the three previously identified MDS-UPDRS III trajectories into a binary outcome to facilitate algorithm implementation and clinical interpretation. Group 1 (slow progression) was retained as the “slow” class, whereas Group 2 (moderate progression) and 3 (rapid progression) were merged to form a single “accelerated progression” class. The dataset comprised features such as age, sex, BMI, DaTscan striatum, MCI status, serum glucose levels, albumin, age at onset of symptoms, baseline MDS-UPDRS III score, gene-subtype of PD, 15-item Geriatric Depression Scale (GDS), Epworth Sleepiness Scale (ESS), and Rapid Eye Movement Sleep Behavior Disorder Questionnaire (RBD), and State–Trait Anxiety Inventory (STAI) total score. Initially, we utilized the Boruta algorithm for feature selection to identify key variables from this pool. Following the selection of significant features by Boruta, we constructed predictive models using these variables. Sporadic missing values in the feature matrix were handled by a training-set–specific simple imputation strategy: continuous variables were replaced with the training-fold mean, and categorical variables with the training-fold mode; these statistics were recalculated within each cross-validation split to prevent data leakage. To ensure robustness, models were trained with a phased integration framework involving human-provided data followed by machine processing for adaptive model architecture. We performed 5-fold cross-validation and utilized grid search for hyperparameter optimization. The optimal model was chosen based on the maximum Area Under the Receiver Operating Characteristic Curve (AUC). Random Forest (Wright and Ziegler, 2017; Probst et al., 2019) emerged as the best-performing model, which was further subjected to hyperparameter tuning and 5-fold cross-validation to ensure optimal performance and reliability. The effectiveness of the Random Forest model was assessed by comparing AUC values and SHAP beeswarm plots to evaluate the contribution of each feature to the model’s predictions.

All statistical analyses were conducted using R version 4.2.2 and the Free Statistics platform version 2.1 (Beijing Free Clinical Medical Technology Co., Ltd.), with results considered significant at a two-sided *p* value < 0.05, ensuring the transparency and reproducibility of our findings.

## Result

### Latent class trajectory analysis

**Figure 2:**
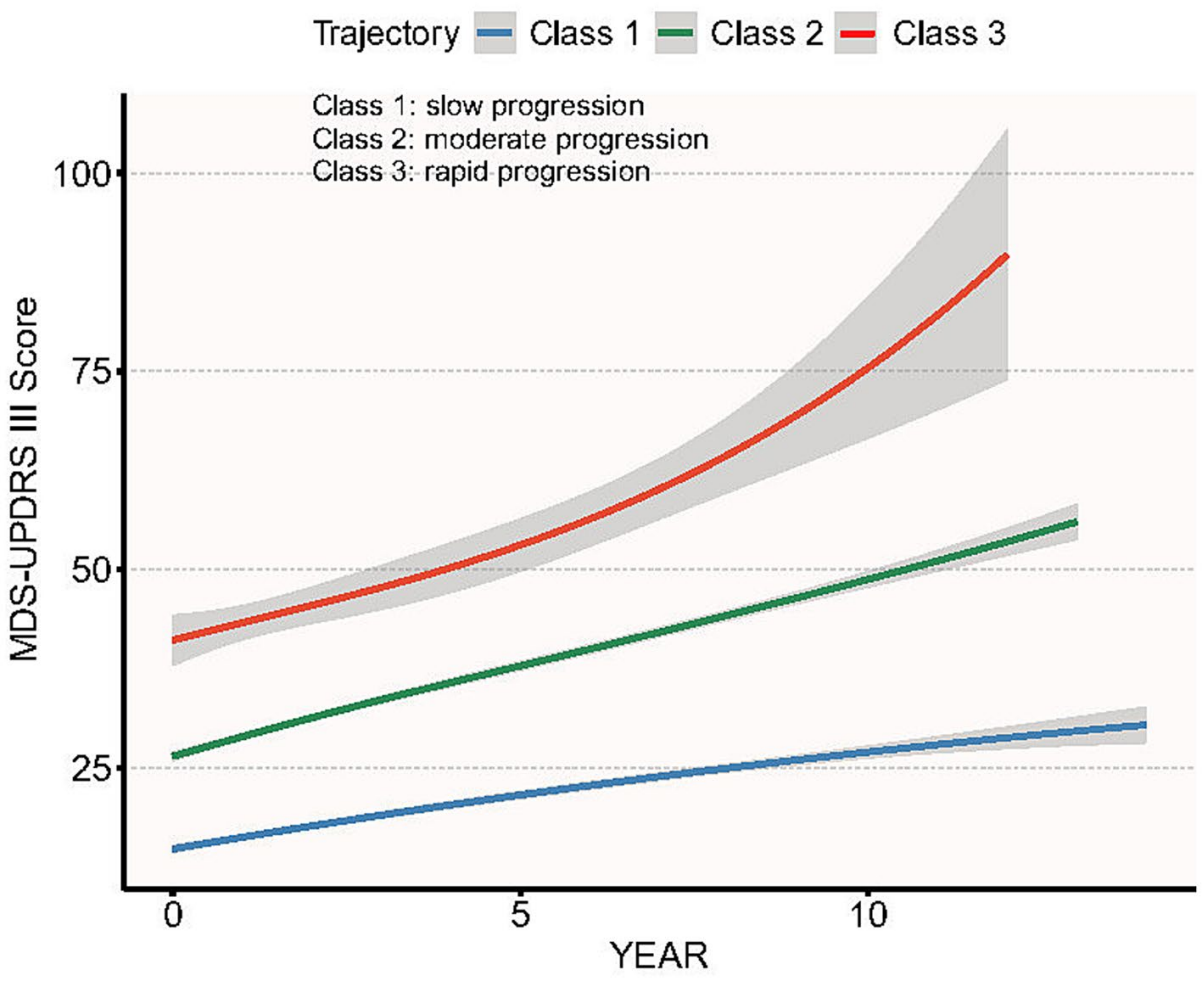
Three distinct longitudinal trajectories of MDS-UPDRS III scores identified by latent class trajectory analysis.

The results of latent class trajectory model construction are presented in Supplementary Table 1. It presented the results of BIC testing, posterior probabilities and classification proportion. For the five evaluated models, only the three-class model exhibited the best fit with the lowest BIC meanwhile the posterior probabilities were above 0.70 and the sample size in each class was above 5%. Thus, the three-class model was chosen as an optimal model. Figure 2 presents the three distinct trajectories of the longitudinal changes in MDS-UPDRS III score. Trajectories 1 to 3 account for 38% (461), 55.9% (679), and 6.1% (74) of the total sample, respectively. The variation of the three trajectories can be interpreted as: Trajectory 1, slow progression; Trajectory 2, moderate progression; Trajectory 3, rapid progression.

### Baseline characteristics of the study population

A total of 1,214 PD patients were ultimately included in the study following the trajectory analysis (Table 1). The majority of the patients were White (93.5%) with a mean age of 62.8 ± 9.7 years. The study cohort consisted of early-stage PD patients. Specifically, the distribution according to the Hoehn and Yahr (H&Y) stage was as follows: 0.3% (*n* = 3) at stage 0, 33.9% (*n* = 397) at stage 1, 64.0% (*n* = 749) at stage 2, and 1.9% (*n* = 22) at stage 3.

**Table 1:** Baseline characteristics of Parkinson’s disease patients stratified by MDS-UPDRS III score progression trajectories.

Variables	Total (*n* = 1,214)	Slow progression (*n* = 461)	Moderate progression (*n* = 679)	Rapid progression (*n* = 74)	*p*
Age (years), Mean ± SD	62.8 ± 9.7	60.9 ± 9.5	63.9 ± 9.6	63.8 ± 10.7	< 0.001
Sex, *n* (%)					0.405
Female	466 (38.4)	188 (40.8)	251 (37)	27 (36.5)	
Male	748 (61.6)	273 (59.2)	428 (63)	47 (63.5)	
BMI (kg/m^2^), Median (IQR)	26.9 ± 4.9	26.4 ± 4.5	27.1 ± 4.8	27.7 ± 7.2	0.019
Race, *n* (%)					0.001
White	1,129 (93.5)	442 (95.9)	624 (92.4)	63 (87.5)	
Black	20 (1.7)	2 (0.4)	12 (1.8)	6 (8.3)	
Asian	16 (1.3)	3 (0.7)	13 (1.9)	0 (0)	
Other	43 (3.6)	14 (3)	26 (3.9)	3 (4.2)	
PD onset age (years), Mean ± SD	59.9 ± 10.2	58.3 ± 9.9	61.1 ± 10.1	60.1 ± 11.1	< 0.001
Gene-subtype of PD, *n* (%)					0.013
Sporadic PD	912 (75.1)	341 (74)	525 (77.3)	46 (62.2)	
Familial PD	302 (24.9)	120 (26)	154 (22.7)	28 (37.8)	
H-Y, *n* (%)					< 0.001
0	3 (0.3)	1 (0.2)	1 (0.2)	1 (1.4)	
1	397 (33.9)	248 (55.9)	144 (21.9)	5 (7.1)	
2	749 (64.0)	193 (43.5)	499 (76)	57 (81.4)	
3	22 (1.9)	2 (0.5)	13 (2)	7 (10)	
MDS-UPDRS I score, Median (IQR)	6.0 (3.0, 9.0)	5.0 (2.0, 7.0)	6.0 (3.0, 9.0)	7.0 (5.0, 12.8)	< 0.001
MDS-UPDRS II score, Median (IQR)	6.0 (3.0, 9.0)	4.0 (2.0, 7.0)	6.0 (3.0, 10.0)	10.0 (6.0, 14.8)	< 0.001
MDS-UPDRS III score, Mean ± SD	22.4 ± 10.1	14.8 ± 5.3	25.8 ± 8.0	39.5 ± 12.9	< 0.001
DaTscan striatal binding ratio, Mean ± SD	1.4 ± 0.4	1.5 ± 0.4	1.4 ± 0.4	1.2 ± 0.4	< 0.001
MCI, *n* (%)					< 0.001
No	1,016 (84.3)	398 (87.5)	568 (84)	50 (67.6)	
Yes	189 (15.7)	57 (12.5)	108 (16)	24 (32.4)	
MoCA, Mean ± SD	26.7 ± 2.6	27.0 ± 2.5	26.7 ± 2.5	25.4 ± 4.1	< 0.001
ESS, Median (IQR)	5.0 (3.0, 8.0)	5.0 (3.0, 7.0)	5.0 (3.0, 8.0)	6.0 (4.0, 10.0)	0.007
RBD, Median (IQR)	3.0 (2.0, 6.0)	3.0 (2.0, 5.0)	3.0 (2.0, 6.0)	5.0 (2.2, 8.0)	< 0.001
GDS, Median (IQR)	2.0 (0.5, 3.0)	2.0 (0.0, 3.0)	2.0 (1.0, 3.0)	2.0 (1.0, 5.0)	0.079
STAI-State, Mean ± SD	32.7 ± 10.3	32.5 ± 10.0	32.5 ± 10.1	35.4 ± 12.4	0.061
STAI-Trait, Mean ± SD	32.7 ± 9.8	32.5 ± 9.7	32.7 ± 9.5	35.0 ± 12.8	0.118
STAI Total Score, Mean ± SD	65.4 ± 18.9	64.9 ± 18.4	65.2 ± 18.4	70.4 ± 24.5	0.061
Serum Glucose (mmol/l), Mean ± SD	5.6 ± 1.2	5.5 ± 1.0	5.6 ± 1.1	5.9 ± 2.1	0.081
Albumin (mmol/l), Mean ± SD	43.7 ± 3.5	44.0 ± 3.6	43.6 ± 3.5	43.5 ± 3.4	0.226

PD, Parkinson’s disease; n, number; BMI, body mass index; race other, includes multi-racial; H-Y, Hoehn and Yahr Scale; DaTscan, Dopamine Transporter Scan; MCI, Mild Cognitive Impairment; MoCA, Montreal Cognitive Assessment; ESS, Epworth Sleepiness Scale Score; GDS, Geriatric Depression Scale Score; RBD, REM Sleep Behavior Disorder Questionnaire Score; STAI, State–Trait Anxiety Inventory. Numbers that do not add up to 100% are attributable to missing data.

### Factors associated with motor progression trajectories

**Figure 3:**
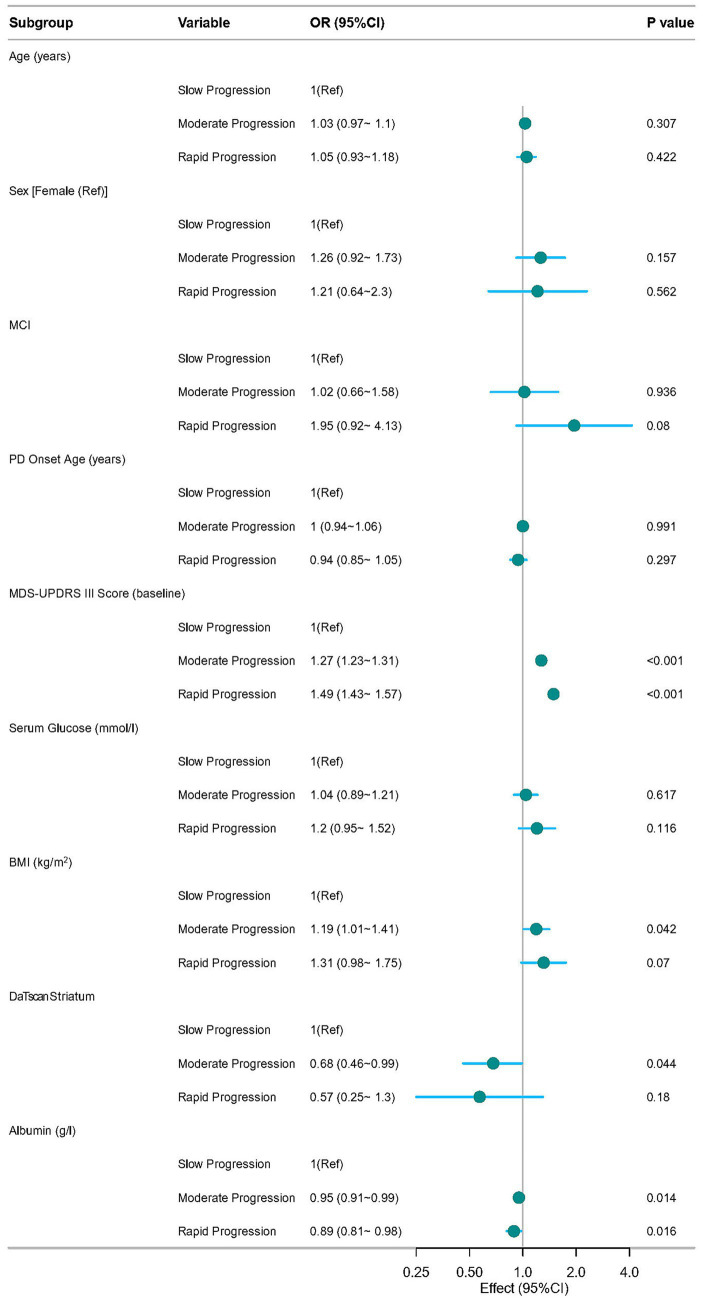
Forest plot of multinomial logistic regression analysis for factors associated with motor symptom progression trajectories.

To investigate the factors associated with the trajectories of MDS-UPDRS III score in PD patients, we conducted multinomial logistic regression analyses, with the results presented in Figure 3. The analysis revealed that baseline MDS-UPDRS III score, albumin, BMI (per 5-unit increase), and DaTscan striatum were significantly associated with the trajectories of disease progression. Every one-point higher baseline MDS-UPDRS III score increased the likelihood of belonging to the Moderate-progression group by 27% (OR = 1.27, 95% CI: 1.23–1.31; *p* < 0.001) and to the Rapid-progression group by 49% compared with the Slow-progression group, after adjustment for all covariates (OR = 1.49, 95% CI: 1.43–1.57; *p* < 0.001). Similarly, each 1 mmoL/L decrement in serum albumin raised the odds of moderate progression by 5% (OR = 0.95, 95% CI: 0.91–0.99; *p* = 0.014) and of rapid progression by 11% (OR = 0.89, 95% CI: 0.81–0.98; *p* = 0.016). Every 5 kg/m^2^ increase in BMI was associated with 19% higher odds of moderate (OR = 1.19, 95% CI: 1.01–1.41; *p* = 0.042), whereas no significant association was observed between BMI and rapid progression. Finally, each 1-unit lower DaTscan striatum reduced the odds of moderate progression by 32% compared with the slow-progression group (OR = 0.68, 95% CI: 0.46–0.99, *p* = 0.044). These findings indicate that baseline BMI, MDS-UPDRS III score, DaTscan striatum, and albumin are significant predictors of the rate of motor symptom progression in PD patients.

### Sensitivity analysis

Sensitivity analyses were conducted to assess the robustness of our findings. First, we excluded all records with missing data (slow progression 450 [37.0%], moderate progression 704 [57.8%], rapid progression 63 [5.2%]) and re-performed the correlation analysis (Supplementary Figure 2). Second, we utilized data from the first 10 years of follow-up to re-run the trajectory (slow progression 601 [42.7%], moderate progression 714 [50.7%], rapid progression 94 [6.7%]) and correlation analyses (Supplementary Figure 3). Both approaches yielded consistent results, confirming the robust associations between the identified predictors and disease progression trajectories.

Specifically, lower albumin levels were associated with an increased likelihood of being in the Moderate Progression group (missing data exclusion: OR = 0.95, 95% CI: 0.91–0.99, *p* = 0.025; 10-year follow-up: OR = 0.95, 95% CI: 0.91–0.99, *p* = 0.016) and a significant trend towards decreased likelihood in the Rapid Progression group (missing data exclusion: OR = 0.87, 95% CI: 0.78–0.96, *p* = 0.007; 10-year follow-up: OR = 0.9, 95% CI: 0.82–0.98, *p* = 0.022) compared to the Slow Progression group. Higher baseline MDS-UPDRS III scores were strongly associated with both the Moderate Progression group (missing data exclusion: OR = 1.27, 95% CI: 1.23–1.31, *p* < 0.001; 10-year follow-up: OR = 1.26, 95% CI: 1.22–1.29, *p* < 0.001) and the Rapid Progression group (missing data exclusion: OR = 1.49, 95% CI: 1.42–1.57, p < 0.001; 10-year follow-up: OR = 1.47, 95% CI: 1.41–1.54, *p* < 0.001) compared to the Slow Progression group. DaTscan striatum was associated with an increased likelihood of being in the Moderate Progression group (missing data exclusion: OR = 0.95, 95% CI: 0.91–0.99, *p* = 0.025; 10-year follow-up: OR = 0.95, 95% CI: 0.91–0.99, *p* = 0.016) and a significant trend towards decreased likelihood in the Rapid Progression group (missing data exclusion: OR = 0.64, 95% CI: 0.42–0.98, *p* = 0.038; 10-year follow-up: OR = 0.65, 95% CI: 0.44–0.94, *p* = 0.022) compared to the Slow Progression group.

For BMI, the association with the Rapid Progression group was inconsistent, showing no significance with missing data exclusion (OR = 1.31, 95% CI: 0.94–1.8, *p* = 0.106), but borderline significance in the 10-year follow-up (OR = 1.33, 95% CI: 1–1.76, *p* = 0.046), whereas the association with the Moderate Progression group remained robust (missing data exclusion: OR = 1.22, 95% CI: 1.01–1.47, p = 0.038; 10-year follow-up: OR = 1.21, 95% CI: 1.02–1.43, *p* = 0.028).

### Machine learning performance

**Figure 4:**
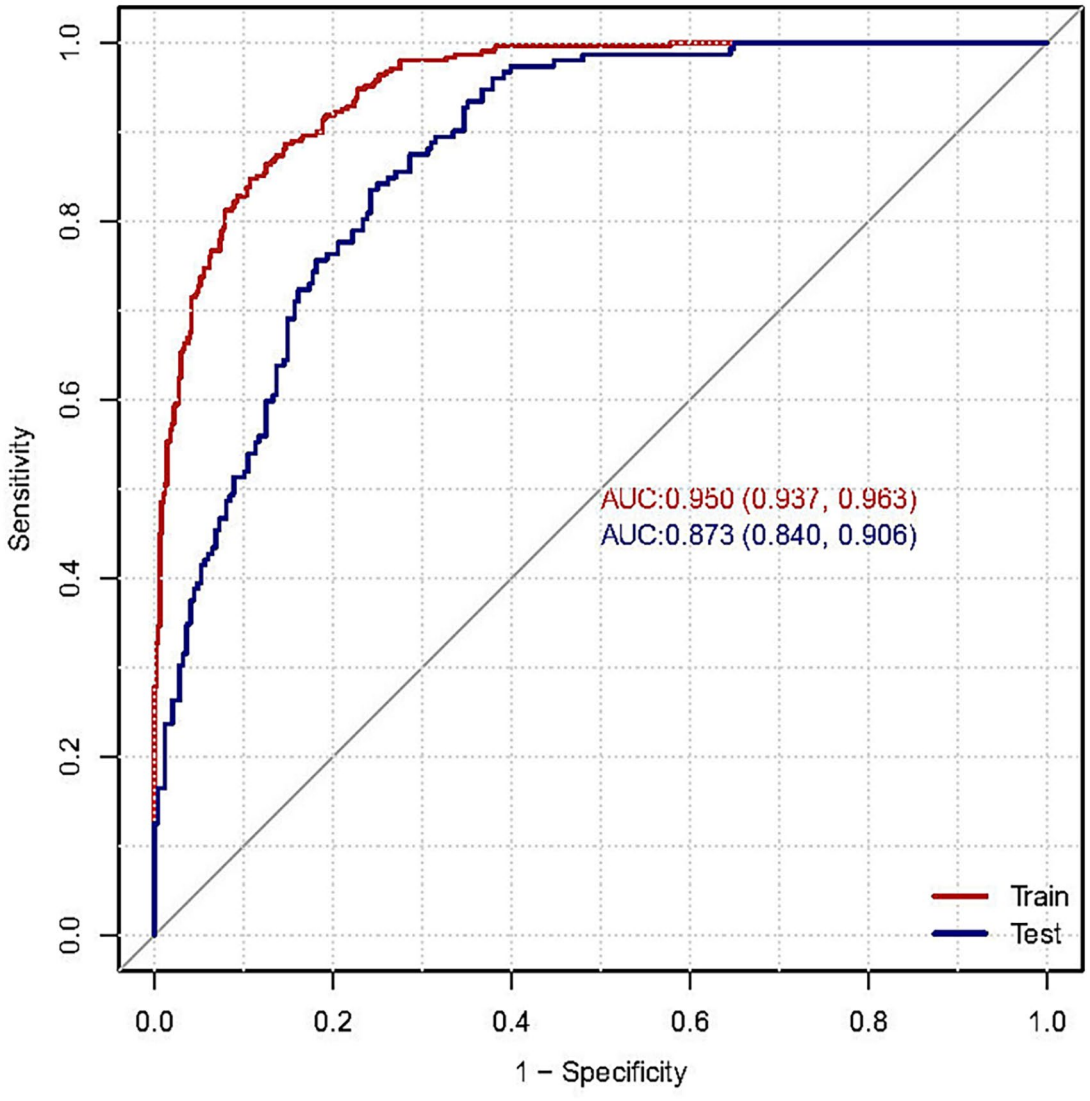
Receiver operating characteristic (ROC) curve of the random forest model on the training and the test set.

**Figure 5:**
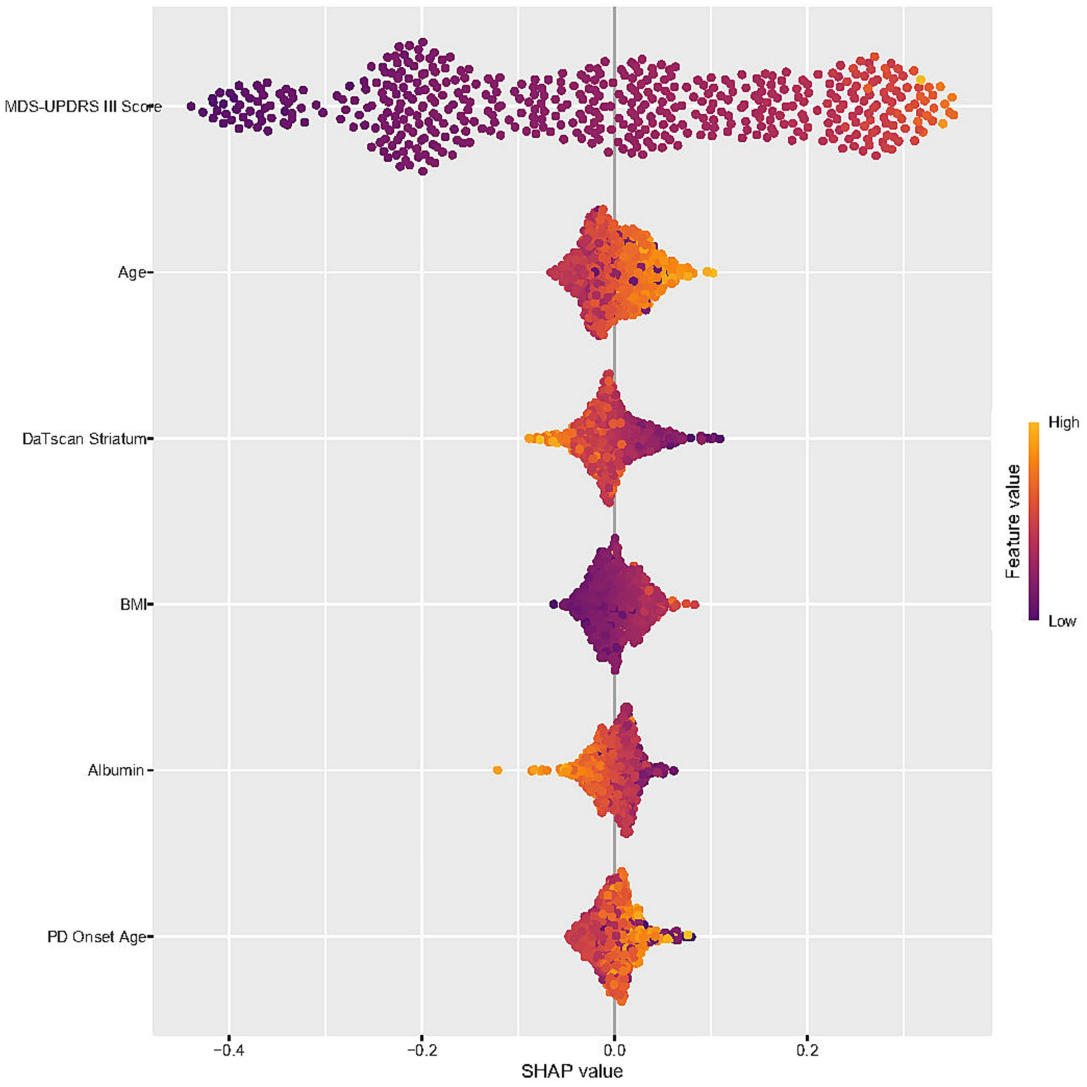
SHAP summary plot showing the importance and direction of effect of each feature on the model’s predictions. Features are sorted in descending order of importance. Each point represents a patient sample. The point’s position on the x-axis represents the SHAP value (impact on the model output), and the color represents the feature value (yellow: high, purple: low).

During the feature selection phase, we utilized the Boruta algorithm to screen all covariates. As illustrated in Supplementary Figure 4, the variables located in the green area, namely age, BMI, mean striatum measure, serum glucose levels, albumin, age at onset of symptoms, and baseline MDS-UPDRS III score, were identified as key factors for the model. These 6 feature variables were ultimately employed to construct the model. When selecting the most effective machine learning method, AUC serves as a pivotal criterion. Consequently, the Random Forest model was deemed optimal. The Random Forest model exhibited an precision of 0.9285, an accuracy of 0.8587, and an F1 score of 0.8806. The AUC value for the training set was 0.950 and test set was 0.873, as shown in Figure 4. It is worth noting that the SHAP-beeswarm plot highlighted the significant roles of baseline MDS-UPDRS III score, albumin, BMI, and mean striatum volume, within the model (Figure 5).

## Discussion

Our study provides valuable insights into the complex interplay between various clinical and demographic factors and the progression of motor symptoms in PD, as measured by the MDS-UPDRS III score. We identified three distinct trajectories of motor symptom progression, highlighting significant associations with baseline MDS-UPDRS III score, albumin, and BMI. These factors emerged as key predictors of different progression patterns. Sensitivity analyses confirmed the robustness of these associations, though the relationship with BMI was partially consistent across analyses. Additionally, machine learning models, particularly the Random Forest model, effectively captured these relationships, achieving high accuracy and AUC values. The most impactful features for predicting the progression of motor symptoms in PD, in descending order of importance, were baseline MDS-UPDRS III score, BMI, age, DaTscan striatum, PD onset age, albumin, and serum glucose. These findings underscore the importance of early assessment of motor symptoms, DaTscan striatum, BMI and serum albumin levels and suggest potential applications for machine learning in predicting disease progression.

A longitudinal study from Singapore reported that lower baseline motor scores were associated with greater progression of motor scores over time (*p* < 0.04) (Reinoso et al., 2015). However, it is important to note that this study included a significant proportion of patients with Hoehn and Yahr (HY) stages greater than 2.5, accounting for 33.28% of the cohort. In contrast, our study included only 1.8% of patients with HY stages greater than 2.5, indicating a difference in disease severity distribution between the two cohorts. Previous research has shown that the rate of motor symptom progression tends to slow down as the disease advances (Louis et al., 1999). This observation may account for the differences observed between our study and the Singapore study. In our study, higher baseline MDS-UPDRS III scores were strongly associated with both the Moderate Progression group and the Rapid Progression group compared to the Slow Progression group. This finding is consistent with previous studies indicating that patients with more severe motor symptoms at baseline are more likely to experience rapid disease progression initially (Chen et al., 2024). Another longitudinal study demonstrated a high correlation between baseline UPDRS scores and the degree of impairment at follow-up (Vogt et al., 2011), further supporting the notion that early motor symptom severity is a significant determinant of disease trajectory.

In PD, serum albumin levels are significantly reduced and have been identified as an independent risk factor for the disease (Wang et al., 2017). Specifically, after adjusting for potential confounders, lower serum albumin levels are associated with an increased risk of severe motor complications [OR 0.34 (95% CI 0.14, 0.80), *p* = 0.013] (Sun et al., 2022). Serum albumin is well-known for its antioxidant, anti-inflammatory, and neuroprotective properties. It plays a crucial role in maintaining plasma osmotic balance, facilitating molecular transport through binding with endogenous and exogenous substances, and providing extracellular antioxidant protection (Sitar et al., 2013). These functions are particularly relevant in the context of PD, where oxidative stress and inflammation are key pathophysiological mechanisms. Moreover, serum albumin has been shown to disrupt the catalytic cycle that promotes the self-aggregation of *α*-synuclein, reshaping α-synuclein oligomers and high-molecular-weight fibrils into isoforms with reduced toxicity. This process inhibits the interaction of the N-terminus and central regions of α-synuclein with cellular membranes (Ahmed et al., 2020). Additionally, serum albumin can impede the fibrillation of α-synuclein and mitigate membrane damage caused by α-synuclein (Kakinen et al., 2018), significantly reducing α-synuclein aggregation at physiologically relevant human serum concentrations (Bellomo et al., 2019).

Our findings demonstrate that a higher baseline BMI independently increases the probability of following a moderate or rapid motor-progression trajectory rather than a slow one, aligning closely with previous evidence that obesity—though not merely overweight—amplifies the risk of early functional dependency and accelerated motor decline in incident PD (Kim and Jun, 2020). Importantly, that same study also detected a significant link between overweight status and faster worsening, underscoring that an elevated BMI per se can serve as an early red flag for impending progression. Complementarily, Wills et al. (2016) reported that participants with a declining BMI exhibited an extra 1.48-point annual increase in motor UPDRS scores compared with those whose BMI remained stable, whereas individuals with rising BMI showed a modest annual improvement of 0.51 points. Nevertheless, their multivariable model did not adjust for BMI at randomization, and the declining-BMI subgroup had the highest obesity rate at baseline, making it difficult to disentangle the intrinsic effect of weight loss from the confounding influence of initial adiposity. Taken together, the two lines of evidence converge on a “BMI two-phase” framework: baseline obesity sets the stage for a more aggressive onset trajectory, whereas subsequent weight loss acts as a disease-course accelerator. While some studies have reported that patients with PD are often overweight or obese at baseline (Barichella et al., 2003; Morales-Briceño et al., 2012), others have demonstrated that BMI tends to decrease over the course of the disease (Uc et al., 2006; Song et al., 2021; Henderson et al., 2025). Future work should therefore prospectively model both baseline BMI and its longitudinal change as joint predictors to definitively test the “two-phase” hypothesis and guide personalized, BMI-targeted interventions aimed at slowing motor progression in PD. In sensitivity analyses limited to 10-year follow-up or missing data exclusion, BMI retained ORs > 1 for rapid progression but crossed the significance threshold, plausibly reflecting reduced event numbers and loss of power rather than a true null effect. Longer, larger, and ethnically diverse cohorts with high-frequency weight measurements are needed to confirm the magnitude and time-dependence of BMI-related motor risk.

Despite the insights provided by this study, several limitations with important clinical implications should be acknowledged. First, the model was developed and validated exclusively within the PPMI cohort, which comprises predominantly early-stage Parkinson’s disease patients (baseline HY stages I–III) and is ethnically homogeneous (93.5% White). Therefore, its applicability to patients with moderate-to-advanced disease (HY stages IV–V) and to more diverse populations—who may exhibit distinct motor phenotypes—remains unestablished. Second, the absence of external validation in independent, real-world clinical settings limits confidence in the model’s generalizability. Routine practice often involves variable assessment protocols and heterogeneous treatment regimens, factors not fully captured in the research environment. Third, although multiple imputation was used to handle missing data, certain potential confounders—such as detailed dopaminergic medication schedules and other symptomatic treatments—were omitted from the model to avoid overfitting. Their influence on motor progression thus remains uncertain. In summary, while the model demonstrated good internal predictive performance (AUC = 0.85), its current clinical utility is largely confined to prognostic stratification and research enrichment in early-stage, well-characterized cohorts. Caution is advised regarding its deployment in advanced-stage or community-based populations until prospective external validation confirms its robustness in real-world clinical practice.a.

## Conclusion

This study identified three distinct trajectories of motor symptom progression in Parkinson’s disease and established baseline MDS-UPDRS III score, serum albumin level, DaTscan striatum, and BMI as significant predictors of progression rate. Machine learning models further confirmed the central role of these factors in predicting disease trajectories. Our findings indicate that early assessment of motor severity, nutritional status, and body weight metrics can help identify patients at high risk of rapid progression, supporting personalized disease management. Future research should focus on validation in multiethnic cohorts, incorporation of longitudinal measures and treatment data to enhance the clinical applicability of predictive models.

## Data Availability

Publicly available datasets were analyzed in this study. This data can be found at: https://www.ppmi-info.org/access-data-specimens/download-data, Parkinson’s Progression Markers Initiative (PPMI) Data Repository Accession identifier: RRID: SCR_006431.

## Footnotes

1
https://www.ppmi-info.org/access-data-specimens/download-data

## Supplementary material

SUPPLEMENTARY FIGURE 1 Machine learning analytical pipeline for predicting Parkinson's disease motor progression trajectories.

SUPPLEMENTARY FIGURE 2 Forest plot of multinomial logistic regression analysis for sensitivity analysis (excluding all records with missing data).

SUPPLEMENTARY FIGURE 3 Forest plot of multinomial logistic regression analysis for sensitivity analysis (using the first 10 years of follow-up data).

SUPPLEMENTARY FIGURE 4 Results of feature selection using the Boruta algorithm. features in green boxes are confirmed as important predictors; features in blue boxes are tentative; features in red boxes are rejected.
