# Supplementary figures and images for: Factors associated with longitudinal MDS-UPDRS III score trajectories in early-stage Parkinson’s disease
Source: Front Neurosci. 2026 Feb 20;20:1759090. doi: 10.3389/fnins.2026.1759090 (PMC12963062; doi:10.3389/fnins.2026.1759090)

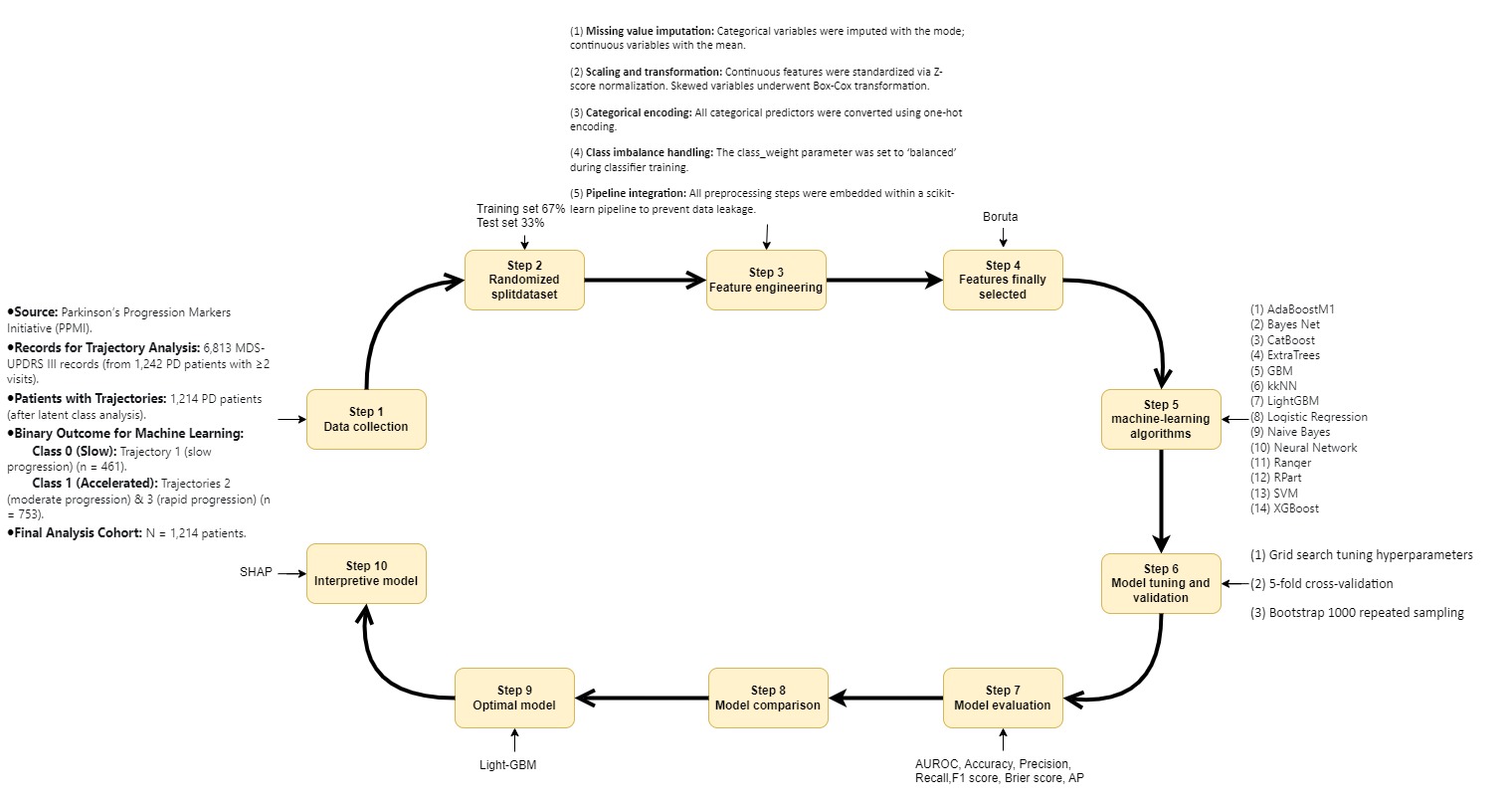

Supplement: SUPPLEMENTARY FIGURE 1 — Machine learning analytical pipeline for predicting Parkinson's disease motor progression trajectories. [file Image_1.JPEG]

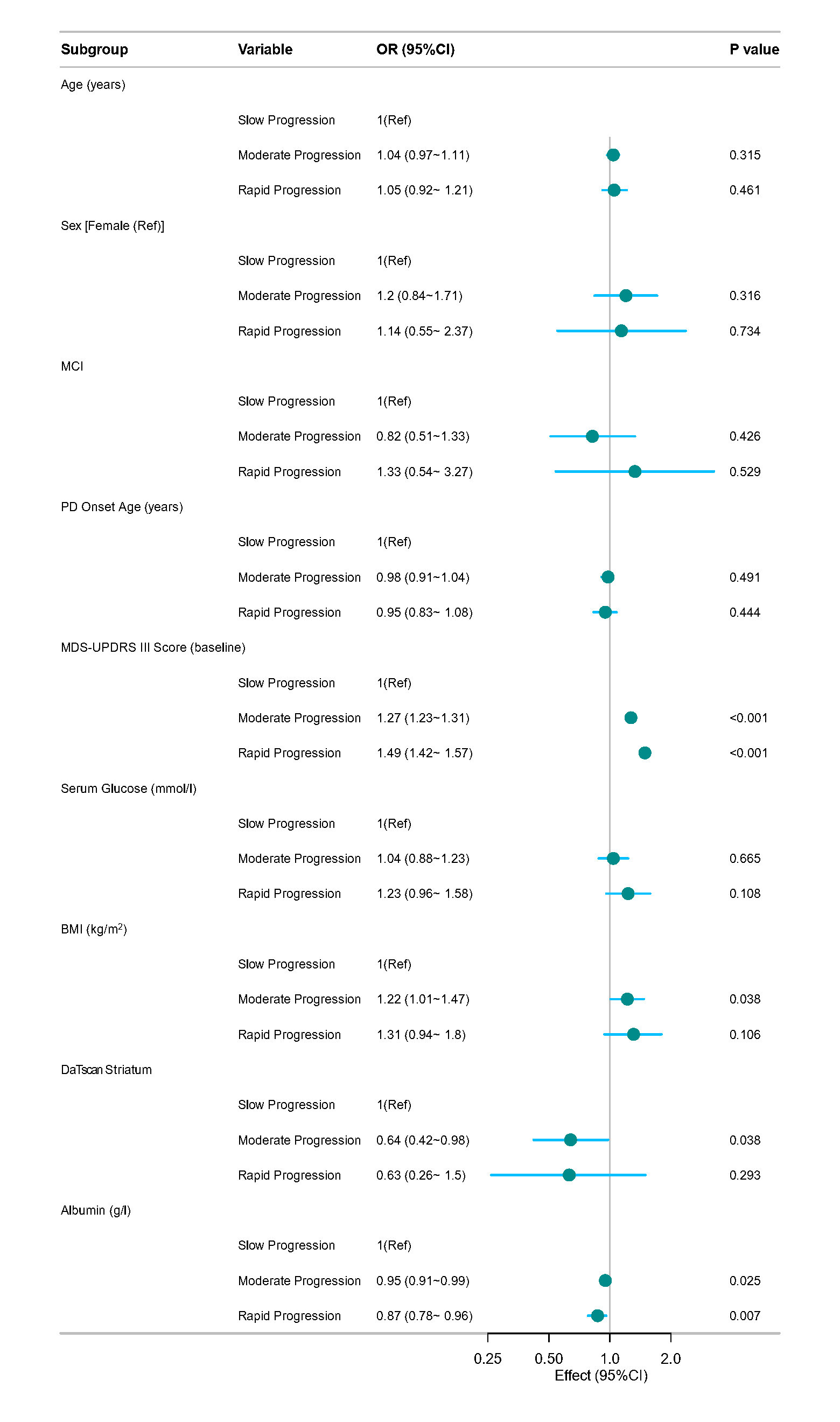

Supplement: SUPPLEMENTARY FIGURE 2 — Forest plot of multinomial logistic regression analysis for sensitivity analysis (excluding all records with missing data). [file Image_2.JPEG]

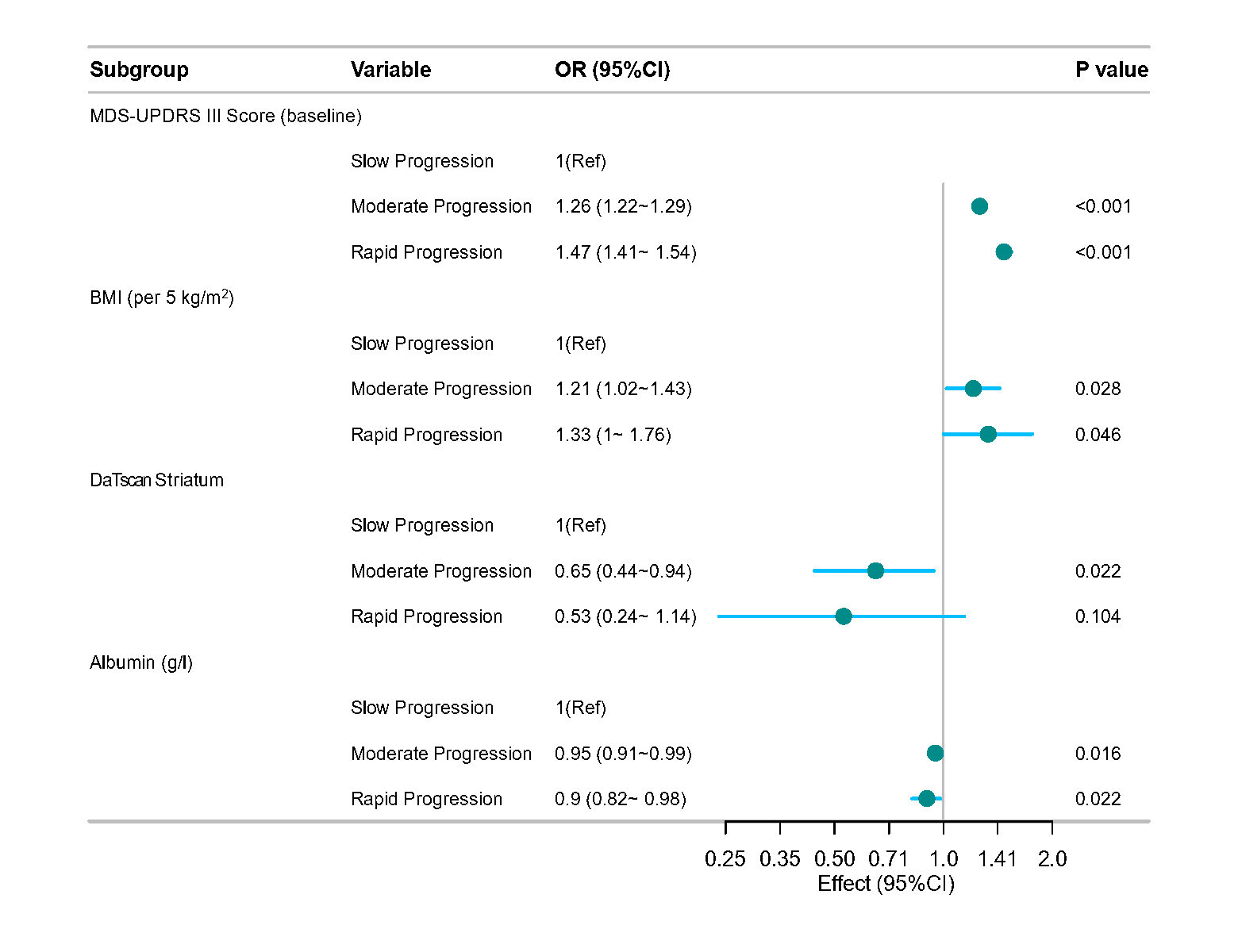

Supplement: SUPPLEMENTARY FIGURE 3 — Forest plot of multinomial logistic regression analysis for sensitivity analysis (using the first 10 years of follow-up data). [file Image_3.JPEG]

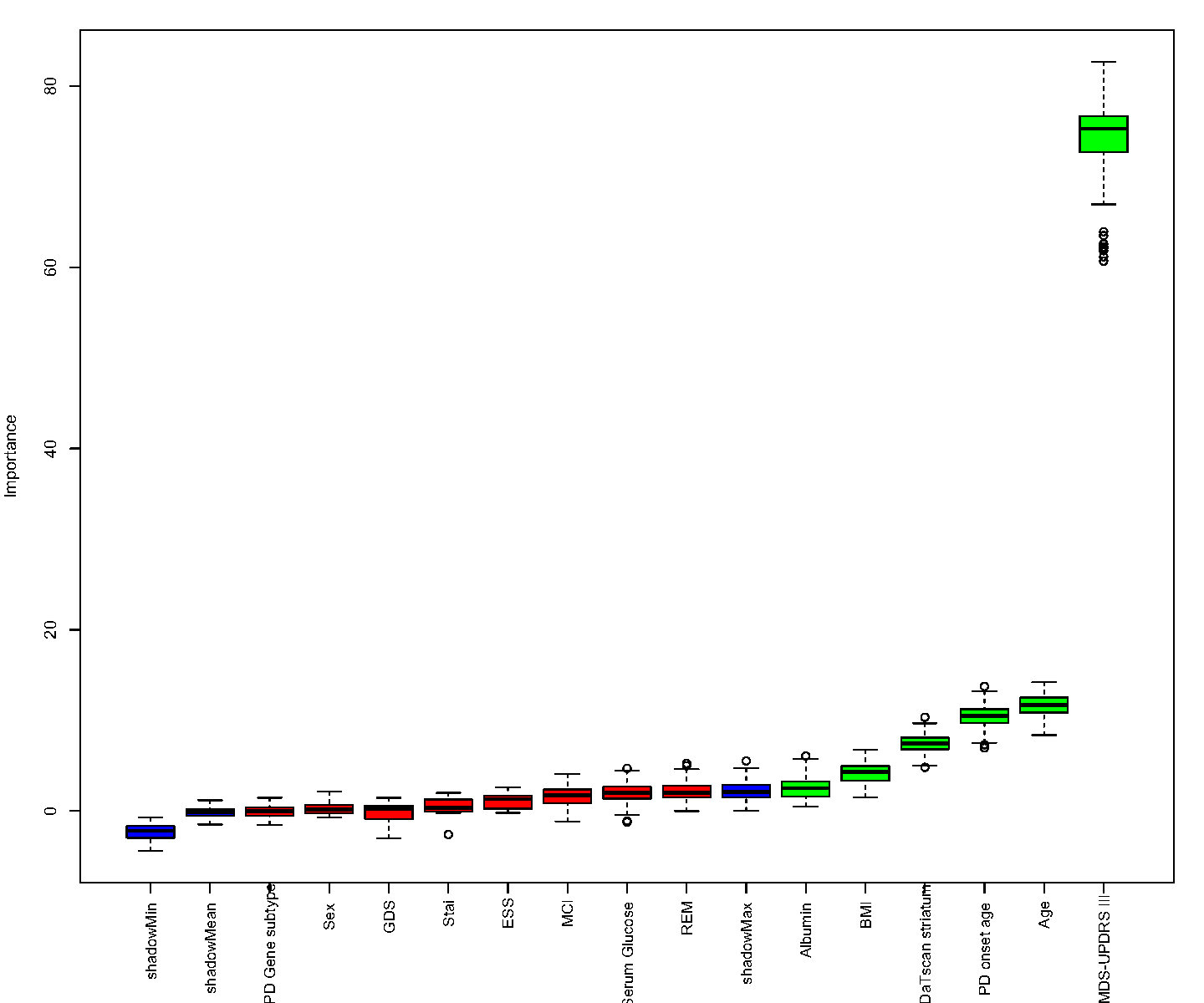

Supplement: SUPPLEMENTARY FIGURE 4 — Results of feature selection using the Boruta algorithm. features in green boxes are confirmed as important predictors; features in blue boxes are tentative; features in red boxes are rejected. [file Image_4.JPEG]
