# Supplementary material for: Factors associated with longitudinal MDS-UPDRS III score trajectories in early-stage Parkinson’s disease
Source: Front Neurosci. 2026 Feb 20;20:1759090. doi: 10.3389/fnins.2026.1759090 (PMC12963062; doi:10.3389/fnins.2026.1759090)
Supplement: Supplementary file 5 [file Table_1.docx]

Supplementary Table 1 Goodness-of-Fit Indices for Latent Class Trajectory Models.

| G | loglik | conv | npm | AIC | BIC | SABIC | entropy | ICL1 | ICL2 | %class1 | %class2 | %class3 | %class4 | %class5 | Posterior.probability1 | Posterior.probability2 | Posterior.probability3 | Posterior.probability4 | Posterior.probability5 |
| --- | --- | --- | --- | --- | --- | --- | --- | --- | --- | --- | --- | --- | --- | --- | --- | --- | --- | --- | --- |
| 2 | -18601.2651 | 1 | 15 | 37232.5301969427 | 37309.055336517 | 37261.4092204254 | 0.390683802845335 | 37821.7831427275 | 37914.2281025681 | 50.0823723228995 | 49.9176276771005 | NA | NA | NA | 0.7274 | 0.8623 | NA | NA | NA |
| 3 | -18581.09301 | 1 | 20 | 37202.186015128 | 37304.2195345604 | 37240.6913797716 | 0.450433588798507 | 38037.1846756816 | 38076.7730022605 | 37.9736408566722 | 6.09555189456343 | 55.9308072487644 | NA | NA | 0.7351 | 0.7748 | 0.7402 | NA | NA |
| 4 | -18571.09563 | 1 | 25 | 37192.1912615449 | 37319.7331608354 | 37240.3229673493 | 0.460413416353855 | 38227.8365284643 | 38315.037857268 | 25.8649093904448 | 45.6342668863262 | 6.17792421746293 | 22.3228995057661 | NA | 0.7056 | 0.6656 | 0.7394 | 0.656 | NA |
| 5 | -18562.38932 | 1 | 30 | 37184.7786487402 | 37337.8289278888 | 37242.5366957056 | 0.474422444791496 | 38364.7326420271 | 38462.6595046093 | 33.3607907742998 | 38.1383855024712 | 2.05930807248764 | 3.62438220757825 | 22.8171334431631 | 0.5973 | 0.7029 | 0.5766 | 0.6931 | 0.631 |
